# Information Consumption, Trust Dynamics and COVID-19 Vaccine Hesitancy among Older Adults: Implications for Health Messaging

**DOI:** 10.3390/vaccines11111668

**Published:** 2023-10-31

**Authors:** Yiyi Wu, Mark Brennan-Ing

**Affiliations:** Brookdale Center for Healthy Aging, Hunter College, The City University of New York, New York, NY 10035, USA; mi708@hunter.cuny.edu

**Keywords:** COVID-19, health messaging, immigrants, low-income populations, older adults, racial and ethnic minorities, trust, vaccine hesitancy

## Abstract

Staying well informed about the evolving COVID-19 pandemic and vaccine recommendations is vital for older adults, especially for low-income older adults, who have been disproportionately impacted by the pandemic. However, the overwhelming infodemic poses a significant challenge, affecting vaccine decision-making. This study explores how a group of predominantly low-income older adults navigate health information and how their trust in information and vaccines evolves throughout the pandemic. Our objective is to provide insights that will guide future public health messaging for this demographic. Analyzing qualitative data from 77 older adults (aged 65 to 94) collected through focus groups and interviews, our findings reveal that participants’ experiences with information overload eroded their trust in authority, leading to vaccine hesitancy. Moreover, the need for a booster has affected belief in vaccine safety and efficacy. As participants lost faith in the media and authoritative sources, they increasingly leaned on personal networks for guidance. These results underscore the urgent necessity for clear, unambiguous ongoing vaccine guidance to restore institutional trust among older adults. Additionally, recognizing the influential role of direct networks in vaccine decisions, integrating care workers, service providers, and peer-to-peer support into health messaging mechanisms could prove valuable.

## 1. Introduction

Since the onset of the COVID-19 pandemic, it is evident that older age is associated with an increased risk of SARS-CoV-2 infection and poorer disease outcomes. Notably, individuals aged 65 and above bear greater odds of both COVID-19-related hospitalization and mortality, accounting for more than 70% of all fatalities in the United States [1]. Moreover, the pandemic has magnified existing health disparities, affecting low-income older adults from racial/ethnic minority and immigrant backgrounds [2]. Compared to non-Hispanic Whites, non-Hispanic Black and Hispanic older adults are more likely to reside in overcrowded conditions, having a higher prevalence of chronic diseases related to poor COVID outcomes, and having poorer health care access [2,3]. Consequently, the COVID-19 mortality risk for older non-Hispanic Black and Hispanic older adults has been nearly twice that of their non-Hispanic White counterparts [4].

The most potent strategy for mitigating the effects of the SARS-CoV-2 virus and curbing its spread is the utilization of the COVID-19 vaccines. The initial vaccine rollout in early 2021, which is now known as the primary series, targeted the original virus strain. As the SARS-CoV-2 virus continued to mutate, booster shots became available to address the continuously emerging variants that heightened disease transmissibility and severity. For older adults, staying informed about the latest CDC guidance and adhering to the evolving vaccine recommendations in a timely manner was particularly critical. Nevertheless, despite robust evidence on the safety and efficacy of the vaccines distributed in the U.S., a concerning level of vaccine hesitancy persisted in the older population, especially among people of color, as evidenced by the disproportionately lower vaccination and booster coverage in these communities even after the vaccines were widely available [5,6,7].

Vaccine hesitancy, defined as, “...a delay in acceptance or refusal of vaccines despite availability of vaccination services”, is considered as one of the greatest threats to public health [8]. Vaccine hesitancy is a complex and context-specific challenge, shaped by an interplay of various factors including individuals’ trust in science, healthcare systems, and governmental institutions; personal political, religious, and sociocultural beliefs; and the accessibility and the perceived risks of the vaccine [9,10]. The challenge of vaccine hesitancy during the COVID-19 pandemic was further complicated by an unprecedented surge in information in tandem with a proliferation of misinformation. The convergence of a global crisis, widespread use of social media, and the entanglement between public health and political ideologies created a so-called “infodemic”, presenting the American public an exceptional challenge in obtaining reliable and accurate vaccine information [11,12].

To combat vaccine hesitancy and encourage uptake, research has underscored the potency of strategic health communication that employs relevant messengers and contextualized messages tailored to the needs of different communities [13,14]. Nevertheless, the state of COVID-related health communication has frequently overlooked disparities in health literacy, cultural relevance, and institutional trust among individuals with lower income and communities of color who were also disproportionately impacted by the pandemic [15,16]. For example, many studies emphasize that mass vaccination media campaigns often fail to resonate with Black Americans who are already distrustful in the medical establishment due to its historical mistreatment of Black individuals in health care settings and biomedical research [17,18]. In addition, certain scientific information containing an overwhelming amount of jargon and intricate data presentation can inadvertently steer the groups with lower education and income levels towards more easily understood yet inaccurate information [19].

Understanding the concerns and needs for information among vaccine recipients is crucial to inform effective public health messaging. The existing literature on the perception of and hesitancy toward COVID-19 vaccination and experiences with health information among communities of color primarily has targeted younger adults. While low-income older adults of color and immigrant older adults often find themselves at the intersection of multiple vulnerabilities, few studies have explored their perspectives, leaving a gap in informed effective messaging for these marginalized populations.

Currently, although the emergency phase of the COVID-19 pandemic has ended, delivering effective health messaging to vulnerable older adults remains a pertinent challenge, as the circulation of COVID-19 variants continue to pose an ongoing threat to public health. Yet, the uptake of bivalent boosters, which provide crucial protection against these variants, remains alarmingly low [20], and the same fate may befall the next generation of vaccines which are best suited to reducing infections and mitigating poor outcomes. The current study aims to address this gap in literature by describing how older adults use health information, particularly older adults of color and immigrants, during an ever-changing pandemic. Our objective is to provide insights that can guide future public health messaging concerning vaccine uptake, as well as other health promotion efforts. More precisely, we seek to examine how participants engaged with health information both before and during the pandemic, identify the change in their trust over time, and discern the obstacles and catalysts influencing their decisions to receive the COVID-19 vaccination.

## 2. Materials and Methods

### 2.1. Source of Data and Procedures

This research was conducted in New York City, in partnership with a leading senior service provider in the city. We conducted focus groups and interviews concerning health messaging and individuals’ experiences with the COVID-19 vaccines. Available information during the development phase of this study suggested that vaccine hesitancy and uptake varied by race and ethnicity. Our recruitment targeted six prominent linguistic/demographic groups of older adults in New York City, including U.S.-born Blacks/African Americans, Spanish-speakers, Russian-speakers, Chinese-speakers, U.S.-born Whites, and West Indian-born Blacks.

Recruitment and data collection took place between October 2021 and January 2022. In collaboration with the organization’s vaccine coordinator, we organized thirteen tabling events at the organization’s various service sites to recruit, screen, and schedule potential participants. The tabling events were specifically designed to assist the recruitment of this research, strategically placed at the entrance of the organization’s residential buildings and activity centers. In addition, the organization’s staff member helped to post recruitment flyers in the languages (English, Spanish, Mandarin Chinese, Russian) predominantly spoken at specific senior program sites. Prospective participants underwent a screening process to assess their eligibility based on the following criteria: (1) being 65 years of age or older, (2) capable of engaging in a 90-min in-person focus group discussion or telephone interview, (3) willing to discuss COVID-19 vaccination, and (4) able to converse in English, Spanish, Russian, or Mandarin Chinese. Although the reception of the organization’s service was not a criterion, most of the prospective participants who stopped by the tabling events and contacted us after seeing the posters were service recipients, as they were the only ones able to navigate freely at those sites.

Although utilizing focus groups is our preferred approach for data collection to harness the benefits of group interaction, we also accommodated individual interviews when deemed necessary. Interviews were arranged for individuals who met the eligibility criteria but encountered issues with the convenience of the focus group’s time or location, or when in-person gatherings were precluded due to COVID-19 mitigation protocols. Each participant provided informed consent upon the beginning of the interview and group discussion. Our research materials, including informed consent, were accessible in all four languages: English, Russian, Spanish, and Mandarin Chinese. To ensure effective communication, our research staff who were either native speakers or individuals with exceptional proficiency in the three non-English languages facilitated focus groups and interviews with Spanish-, Russian-, and Chinese-speaking participants. Focus groups and interviews were audio recorded with participants’ consent. The study protocol gained approval from the City University of New York Institutional Review Board. To compensate our participants for their time, each of them received a USD 20 gift card.

### 2.2. Participant Demographics

Overall, we conducted six focus groups and fifteen individual interviews, cumulating in a total of seventy-seven participants. The majority engaged in focus groups (n = 62). The number of participants in each focus group varied, ranging from 4 to 13 individuals. The U.S.-born Blacks comprised the largest group (n = 22), followed closely by individuals identifying as Hispanic/Latinx (n = 21). Other demographic groups included Russian-speakers (n = 11), Chinese-speakers (n = 9), U.S.-born Whites (n = 8), and West Indians (n = 6) (Table 1). Participants were predominantly women. All of the Russian-speaking participants, Chinese-speaking participants, West Indian participants and a few in the Hispanic group were first-generation immigrants. Of note, most Chinese-speaking immigrants indicated during the interviews that they had migrated to the U.S. later in life.

The participants’ ages ranged from 65 to 94, with the majority in their 70s. The living arrangements varied, with most participants living alone, while the rest lived with either children or spouses. While we did not collect individual income data from each participant, it is worth noting that the partnered senior service provider primarily serves low-income older adults. The provider’s income eligibility threshold is set at below 50% of the median household income in the metropolitan area. Hence, most of our research participants were low-income.

### 2.3. Qualitative Analysis

The group discussions and interviews were conducted using an interview guide that covered a wide range of topics. Interview questions included sources of health information before and after the pandemic. Participants were also asked about their opinions on the COVID-19 vaccines and health messaging campaigns and prompted to discuss the evolution of their perspectives over the course of the pandemic. Additionally, we inquired about their general pandemic experiences and sought their recommendations for improving the effectiveness of health messaging. Each focus group and interview lasted around 90 min. All the recordings and interview notes were encrypted. To transcribe English-language audio files, we employed Descript software (version 41.1.0, https://www.descript.com/, accessed on 31 August 2023). For non-English files in languages including Chinese, Russian, and Spanish, we hired a third-party vendor to perform translation and transcription. The Atlas.ti 22 qualitative software package (https://atlasti.com/, accessed on 31 August 2023) was used to facilitate our analysis. 

We took an inductive thematic analytic approach to these narrative data. Based on the interview guide, an initial codebook was developed. Subsequently, the codebook underwent an iterative refinement process following the initial coding of each focus group and interview. Throughout the coding process, the research team met regularly to deliberate on preliminary findings and make necessary coding adjustments. Through these collaborative efforts, a final codebook was established. Utilizing the final codebook, two members of the research team coded each transcript independently. Any differences in coding were resolved through collaborative discussions.

After the codes were refined, we formed code groups for codes with related content. We then used an axial coding approach to examine the associations between the code groups and codes around trust/distrust and attitudes change over time and codes related to information sources, information inconsistency, misinformation, and global perspectives. Through this process, we identified five themes which are discussed below.

## 3. Results

To provide a context to our findings, it is important to note that the interviews and focus groups took place during the pandemic period when the delta variant was the predominant circulating strain, and the first round of boosters had been strongly advised by the CDC and available for months. During the course of data collection, the omicron variant also emerged. All participants had already been vaccinated with the primary series, while a few got their first booster dose. Additionally, many participants reported receiving a routine flu shot and vaccines against pneumococcal pneumonia and shingles. While the prevailing sentiment towards the COVID-19 vaccine was generally positive in this population, a significant number mentioned encountering moments of vaccine hesitancy, particularly regarding boosters. Our findings primarily focus on participants’ narratives of hesitancy, offering insights into the implications for ongoing vaccine recommendations and public health messaging.

Five main themes emerged in our analysis: (1) information overload eroded trust in authority and consequently seeded vaccine hesitancy during the primary series rollout; (2) encounters with misinformation led to vaccine uncertainty among only a few; (3) the need for a booster tarnished people’s previous belief in vaccine efficacy and safety; (4) direct personal social networks emerged as a strong facilitator of decision making; and (5) immigrant participants shared unique experiences with information and rules (Table 2).

### 3.1. Information Overload Eroded Trust in Authority and Consequently Seeded Vaccine Hesitancy during the Primary Series Rollout

When asked about their usual way of consuming health information prior to the pandemic, participants reported prioritizing information disseminated by entities regarded as “authorities”. There were healthcare authorities, which included their own healthcare providers, well-known health organizations, such as the CDC, and influential medical experts who have an active voice in the media (e.g., Dr. Anthony Fauci). Another commonly identified source was governmental authorities, namely government representatives, websites, and social media accounts. Then came media authorities, including TV channels, newspapers, and other social media.

Following the onset of the COVID crisis, many participants were overwhelmed by the relentless flow of information. Much of the information received was perplexing, delivering consistently discordant and changing messages about assessments of pandemic evolution, contagion mitigation measures, and vaccine protocols. This eroded participants’ trust in what they used to perceive as authorities. Consequently, participants across all demographic groups declared having doubts about the efficacy and the necessity of the vaccine during its initial rollout.

*Well, now everyone is listening, watching [TV], and it’s impossible to watch anymore. I wanted to understand what was going on. An extraordinary event in our lives. But I still caught this from a sea of information, because many doctors and some competent people said that if a person has been ill and antibodies have formed, then this is the most reliable protection against the consequences of the next illness.*

(Russian-speaking Participant)

Despite the influx of incoming information, a common sentiment among participants was that few sources managed to deliver clear, accessible, and convincing messages.

*I don’t think they really explained to you what you would be agreeing to have injected into your body…what am I going to be?*

(U.S.-born Black Participant)

*You get something on this station and on another one you get something else, then another one you get something else, you know? You, you can’t get, which is the right one. Some of them, some of the articles make sense. And you have to, you have to read between the lines, you know.*

(West Indian Participant)

To a few U.S.-born Black older adults, mistrust in medical authority also predates the pandemic:

*We have to remember that years ago when minorities were used as the guinea pigs, that’s in the back, that was in the back of a lot of people’s, in terms of hesitancy to take this vaccine. I don’t think that there’s a knowledgeable minority person that didn’t think of the Tuskegee experiment.*

To some others, the turbulent information landscape took a final toll on their confidence, even with the most reputable institutions such as the CDC. One U.S.-born White older adult shared their frustration as: “Dr. Fauci is useless, cause every day it’s something different with them and the CDC. He says he gets it from the CDC”.

Consequently, the combination of lack of information clarity and perceived vulnerability of older adults during the pandemic also made some begin to question the trade-off between the benefits and risk of the vaccines. After being advised by her doctor to receive the vaccine in early 2021, one Chinese-speaking participant did not receive her initial two doses until the summer of 2021. She explained her initial refusal as: “I had doubts initially, too, because I had never heard of this vaccine. At that time, it was said that the older the person is, the greater the risk is with the vaccine”.

### 3.2. Encounters with Misinformation Led to Vaccine Uncertainty among Only a Few

Established media channels such as television and newspaper were more prevalent sources of information, whereas a smaller yet considerable amount participants integrated social media platforms, such as Facebook, Whatsapp, and Wechat, into their daily news consumption. Among the social media users, many of them recounted instances of coming across information that they were able to discern as inaccurate. One Russian-speaking participant shared: “Do you know what the Internet spreads? Those who have been vaccinated will live only 2–3 years! After that, they will definitely die! Such nonsense!” Another Spanish-speaking participant shared: “I didn’t want to know all those negative things. Uh, they kept sending me, ‘Don’t get vaccinated, because you’ll grow four ears. Your nose will grow.’ What’s wrong?”.

Recognizing various aspects of the pandemic became battlegrounds for political agendas, a few participants also reflected on how such a polarized environment can distort health communication objectives that fuel biased information that only aligns with people’s political viewpoints:

*The misinformation that was put out there, it was a lot of, and it was all depending upon the sources from where it came [from], because as someone pointed out, there was a lot of politics that was played into that as well.*

(U.S.-born Black Participant)

Nevertheless, a few others held a more ambivalent stance towards misinformation and exhibited a degree of receptiveness to it, which could potentially influence their ongoing vaccination decisions. One West Indian participant revealed a sense of uncertainty and resignation: “I heard things about whether the shot messes with your DNA. But by then I had taken my first and second shots. So, if that was the truth, it’s already messed with mine”.

In some cases, some individuals began to subscribe to politicized narratives and logics of misinformation. One U.S.-born Black participant said: “Well, at the time I am thinking about Trump was in the office. And I don’t know if he made the vaccine or what. And I was saying if it had anything to do with him, I don’t know”.

### 3.3. The Need for a Booster Tarnished People’s Previous Belief in Vaccine Efficacy and Safety

After the dissemination of the primary series of the COVID-19 vaccines, the CDC issued two boosters amid the delta and omicron variant waves between mid-2021 and early 2022. Although the two booster doses shared the same mRNA technology as the mostly widely used original vaccines, participants were observed to express more suspicion and hesitancy towards boosters. At the time of the interviews, some individuals expressed their desire to delay their booster reception which had been available for months and others explicitly refused to get the booster. Even among those who had received their third dose, many showed their deep concerns and reluctance to get additional COVID vaccine shots. 

Upon the introduction of the first booster, there was again an evolving series of recommendations about booster timing and age eligibility. In addition, a debate on whether it was safe and effective to mix different vaccine brands ignited, creating further controversy. This led to more confusion and inconsistencies in media narratives and delayed some participants’ reception of boosters during a time when New York City was hit hard by rampant new variants and breakthrough infections.

*I was waiting to get the same one that Johnson and Johnson. So I haven’t heard that they have Johnson and Johnson. I just heard on television that you can take, even though I said I don’t listen to the television: I do listen to it a little bit. They said that you could take anything, you could take anyone. But I think to myself, I think if I’m going to take it, I want to take the same Johnson and Johnson. Like I said, it’s too many conflicts. And one day they say, yes and the next day they say no. I’d like to keep it in the same family. So that’s what I’m waiting for.*

(West Indian Participant)

In addition, misinformation continued to spread as the COVID-19 booster program expanded. One U.S.-Born Black participant shared the rise of vaccine skepticism around her immediate circle: “That’s the one that’s going to turn us into a magnet. That’s going to turn us into magnets. That’s what my son said”.

More notably, the rapid rise of breakthrough cases and uncertainties associated with new variants collectively led many to re-evaluate their previous convictions regarding the necessity and effectiveness of the vaccine since the initial vaccine messaging suggested they would durably prevent SARS-CoV-2 infection. One Spanish-speaking immigrant participant asked, “...why is the vaccine there if you are still getting the COVID? I don’t know”. For some participants, the continuous promotion of booster shots shattered their previous expectations and confidence in the efficacy of the initial two vaccine doses.

*We were not worried when we received our first and second doses. However, we were worried when we got our third one. Why? It is because we thought the first and second doses were enough to stop the disease from spreading. We had no choice, so we took the third shot. And now we do not know anymore. Now, they were saying that even if you receive all the doses, you can still get COVID. We are even more scared and do not dare to go outside.*

(Chinese-Speaking Participant)

Given the rapid development of variants, some became frustrated about the never-ending pandemic and were overwhelmed by the amount of vaccines they might have to receive, resulting in vaccine rejection.

*Well, I heard on the news, they said they got a fourth vaccine. I was like, oh my God, what the hell are they putting into us? They been putting all these needles in us…I figured I was going to take the third one. But then when they said they got a fourth one, I don’t know.*

(U.S.-born Black Participant)

And there was the following exchange in one of the Russian-speaking focus groups:

[First Participant] *So, the vaccine doesn’t work*. [Second Participant] *Well, you can’t say that*. [First Participant]: *Because COVID mutates every time*. [Third Participant] *COVID mutates every time. Then we made a vaccine against one type of COVID, now the second type*. [Fourth Participant] *It’s not the second one, it’s just added now, the mutation is happening*. [Third Participant] *It mutates… That vaccine doesn’t work anymore. Thirty letters of the alphabet. Do you understand this? Thirty letters... tomorrow they will come up with what else is alpha, beta.*

Despite participants’ frustration with the repeated booster shots, most participants had a framework for repeated vaccinations as they reported having received seasonal flu shots in the past.

### 3.4. Direct Personal Social Networks Emerged as a Strong Facilitator of Decision Making

Evident from participants’ narratives was the extent to which people believe in institutionalized information and that mediated forms of communication were limited during the COVID pandemic. Instead, the participants’ narratives underscored a heightened reliance on the messages conveyed within their personal social networks, which emerged as a particularly impactful facilitator for their decision to get vaccinated.

Across all demographic groups, individuals described engaging in multiple conversations with families and friends. Among these participants, those who already had received the vaccine wielded significant influence. For instance, a scenario was recalled by one U.S.-born Black participant who detailed how her initial reluctance to be vaccinated was countered by persistent persuasion from her children and nieces. A Chinese-speaking participant with multiple underlying disease conditions was initially worried about the vaccine side effects and was motivated to take the shots upon learning of their peers’ positive experiences with the vaccines.

More notably, beyond families and friends, participants’ direct service providers also play an influential role in shaping their decisions. Since most of the participants were service recipients of a large senior serving organization including residents of senior housing, they recounted receiving check-in calls and receiving health information sheets from the organization staff.

*And then people were just like, oh, you better take it. You better take it. You better take it. Especially the people, as the directors of the centers, I deal with them a lot. They would like for your own safety, think about your own safety. Think about your family, who you are around all the time. You want to be safe. That’s why I took it.*

(U.S.-born Black Participant)

Similarly, one Chinese participant shared: “The staff at our apartments informed us. Since it is a senior apartment, they informed us well. As soon as we saw the notice, we immediately signed up for the vaccine”. It is worth noting that Chinese-speaking participants depended more on the guidance of the staff from the senior service provider than participants from other demographic groups. Since many Chinese-speaking participants migrated to the US later in life, they often lacked established social networks in the U.S. and spoke very limited English. Thus, they regarded senior building staff as primary sources of information and support and hence, had a lot of trust in these organizations. 

Some others also appreciated the companionship and support provided by their home attendants during the pandemic who actively connected them to vaccine information and resources.

*Um, yes, I didn’t want to go through with it. But my aide, she said, mama, if I have to carry [you] on my back, I’m taking you to get a vaccine, because I am going to take mine. I knew I have to go and get yours okay. Then the people from the center called me and they talked to me about it. And so, um, that was it. I said, okay.*

(West Indian Participant)

Harboring skepticism about healthcare representatives in the media, some participants placed more faith in the words of their own healthcare providers with whom they had developed personal relationships with over time.

*If I could explain. Well, I asked my doctor, my primary doctor, I asked him, I said that, is the thing good for us to do vaccine because you’re hearing all sorts of problems. Some of them really be misinformed…And he said to me, he said, take this vaccine, especially in your case. He said, get the vaccine, go take the booster. I obeyed.*

(West Indian Participant)

More remarkably, many older adults also described their efforts to get others including their peers to vaccinate. One Spanish-speaking participant shared their conversation with a friend:

*But you know, I really like to be in your company. I’d like for us to do that, but without your having had the vaccine I’m not comfortable with that. And I’m asking you to do it for you and for me. For both of us. That’s the only way.*

### 3.5. Immigrant Participants Shared Unique Experiences with Information and Rules

The majority of the immigrant older adults consumed information in their native language including channels that are US-based, as well as those originating from their home countries. As shown in the narratives of doubts among some immigrant participants who lived through the crisis in the U.S., immigrant older adults were certainly not immune to the repercussions of the rapidly evolving and politicized pandemic landscape. However, many older immigrant participants were able to harness information beyond the U.S. context, thereby mitigating the impact of constant influx of inconsistent information. For example, during the early months of 2020 when conflicting reports regarding the severity of the virus were bewildering many U.S. born participants, Chinese participants had already gained a clear understanding of the pandemic’s trajectory. This was attributed to insights garnered from media outlets and personal networks from China, where the pandemic initially went rampant. Recalling their experiences from that period, one participant shared: “We Chinese at the time were already wearing masks in the subway…when foreigners [referring to Americans] said there was no need”.

Overall, among the Russian-speaking groups, the Chinese-speaking groups, and individuals hailing from the West Indies, there was a prevailing sentiment about the importance of following and adhering to social norms. Living through the era of the Soviet Union, a time characterized by the establishment of pro-vaccination norms, one Russian-speaking participant explained their decision to take the vaccine as: “How about it…!? I’m a Soviet person. I am all for vaccines”.

Some participants from various countries across the West Indies were perplexed by the ongoing debate on the vaccine mandate in the U.S. and shared: “it was simply what you did and went without saying. [re vaccination] Well, um, I grew up in the West Indies and it was par for the course. We had no say in it and our parents believed in it”.

## 4. Discussion

This qualitative study explored the dynamic process of information navigation and vaccine decision-making among a group of older adults, the majority of whom were low-income, in the context of an ever-changing pandemic. The findings reveal a noteworthy shift in trusted sources of health information among this population after the pandemic outbreak. Prior to this global crisis, participants routinely relied on televised news, newspaper, and health and political authorities as their primary sources of information and held those sources in high regard. However, as the pandemic unfolded, those platforms struggled to deliver a clear consistent message about the pandemic landscape and the COVID-19 vaccines. This challenge was further exacerbated by the proliferation of misinformation and increasingly polarized political climate. Our participants, like many others, were overwhelmed and started losing confidence in these once-trusted sources. Consequently, echoing previous studies, the pervasive experiences with information overload dampened the participants’ intentions to get vaccinated [12,21,22]. Moreover, the older demographic is usually associated with a greater degree of institutional trust in the government and health authorities [23]. However, demonstrated by the outspoken frustration with these authorities among some participants, this study also sheds light on a decrease in institutional trust among older adults, as a result of the pandemic.

Nevertheless, despite their understandable frustration with the chaotic information landscape during the pandemic, many participants demonstrated remarkable resilience in the face of the widespread misinformation. In contrast to past research that suggests demographic factors such as older age, low-income, and belonging to a minority race/ethnicity are associated with a higher tendency to consume and believe in misinformation [24,25], the narratives presented in this study show otherwise. Many participants were aware of current issues, able to discern inaccurate information, and even took further steps to inform others. However, this study also shows the presence of a minority who displayed a degree of receptiveness to misinformation that can potentially influence their vaccination decision. This highlights the continued importance of efforts in combating and ratifying the spread of misinformation.

In line with quantitative reports that demonstrate decreasing booster coverage among older adults [7], this study shows a higher degree of hesitancy, and even refusal, towards the COVID-19 booster even though the boosters had become widely available upon the start of our data collection. The ongoing messaging inconsistencies with booster eligibility and guidelines and spread of misinformation certainly play a role in establishing hesitancy among some participants. More importantly, it is also the initial lack of messaging on the waning immunity, virus mutation, and the need for ongoing immunity boost during the dissemination period of the primary series, which, in the subsequent stages, shattered people’s belief in the vaccine efficacy altogether and caused them to question the value of the booster.

Moreover, as participants gradually lost faith in the credibility of the media and authoritative sources that are often associated with mediated communication, they began to place greater trust in individuals within their personal networks, including their home care aides and service providers whom they share personal relationships with. Such a finding is consistent with past studies that identify social networks as a strong facilitator of vaccination decisions [26,27]. Echoing past research, community-based efforts can play a pivotal role in combating vaccine hesitancy through tailored, localized strategies that address the specific concerns and needs of different groups. More notably, this research also suggests the particular influence possessed by older adults’ peers. In a different paper from this study [26], we explicitly explored extensively on how older adults themselves serve as health advocates and sources of support.

Lastly, this study did not find patterns of trust dynamics and vaccine uptake by demographic groups. Nevertheless, it is worth highlighting that the Russian-speaking groups, the Chinese-speaking groups, and participants from the West Indies suggested that their adherence to the rules and guidelines was the key motivator to get vaccinated.

This study bears certain limitations. Our recruitment materials described our research’s primary focus on attitudes towards the COVID-19 vaccines, which may deter some participants who had not received the vaccines and/or held more negative views towards them. Additionally, the study took place in New York City, where political and sociocultural norms towards vaccination tended to be more positive than other parts of the U.S. Such an environment might have discouraged individuals who strongly opposed to vaccines from participating, as they might have sensed that their viewpoints were less likely to find approval from others. Hence, future research should place a special focus on older adults who had not been vaccinated or reside in regions with low vaccination rates. Additionally, in the case of immigrant participants, the length of time spent in the U.S. was not intentionally gathered. Future research with a focus on immigrants should place particular emphasis on this aspect and elaborate how the duration of their residency in the U.S. shapes their health information consumption patterns and vaccine-related decisions.

The strength of this study lies in the diversity within our research sample, as our research participants came from diverse racial/ethnic and immigration backgrounds. More importantly, this study offers unique insights into the challenges and success surrounding health information navigation, health communication, and crisis management within a predominantly low-income older adult demographic. Given the disproportionate impact of the COVID-19 pandemic on this group, the narratives presented in this research not only shed light on the difficulties they faced but also highlight their remarkable resilience in the face of crisis.

## 5. Conclusions

The findings in this study explored the ways of navigating information and evolving dynamics of trust among a group of predominantly low-income older adults during the COVID-19 pandemic. The waning trust in perceived authorities underlies the urgent need for greater clarity and unambiguity in ongoing guidance to re-establish institutional trust among the older population. The heightened skepticism surrounding booster shots marks the importance of ongoing health messaging to specifically address booster hesitancy and rebrand boosters. Considering older people’s acceptance of other routine vaccine shots, there is a real opportunity to boost the booster by reframing it as part of routine preventative health care. In addition, given the demonstrated effectiveness of older adults’ direct networks in influencing their vaccine decisions, including care workers, service providers, and peer-to-peer support into health messaging mechanisms could be valuable.

## Figures and Tables

**Table 1:** Demographic Characteristics.

Demographic Group	n	%
U.S.-born Black	22	28
Hispanic/Latinx	21	27
Russian speaker	11	14
Chinese speaker	9	12
U.S.-born White	8	10
West Indian	6	8
Total	77	100

**Table 2:** List of codes used in analysis and example quotations.

Theme	Codes Used for Analysis	Example Quotation
Information overload eroded trust in authority and consequently seeded vaccine hesitancy during primary series rollout	Distrust; Information sources (sub codes: news/TV, scientist, doctor/medical professionals, social media, social networks, websites, and senior center’s staff); Inconsistent information/doubt	But when your leaders get on TV, it’s like anybody else. If you were talking to me, trying to convey a message to me and you don’t really seem convicted to it, are you really sure about it? They kept flip-flopping and flip-flopping and what do they know what they’re talking about? (U.S.-born White participant) Some of the concern was that it seemed like they were waffling too, and I say “they”, I’m talking about the medical community. (U.S.-born Black participant) When I want to tell you the truth, there was so much hype about this vaccine that you don’t know what to believe, and what not to believe (Spanish-speaking participant)
Encounters with misinformation led to vaccine hesitancy among only a few	Distrust; Information sources Information sources (sub codes: news/TV, scientist, doctor/medical professionals, social media, social networks, websites, and senior center’s staff); Misinformation	Because even some doctors giving you some misinformation too, ‘cause I heard one. one night and I was seeing, he is a doctor and all against the thing like that, you know? (West Indian participant) They were also saying that they are using this vaccine put chips in you, keep tracking you, all that kind of craziness. (U.S.-born Black participant) In a lab. And it got out of control. So, now, you know, the whole world is getting money for vaccines and others inventing from here and inventing from there. (Spanish-speaking participant)
The need for a booster tarnished people’s previous belief in vaccine safety	Distrust; Booster; Inconsistent information/doubt	We sometimes feel that there is still no end to vaccination. After all, how many new viruses can be prevented? (Chinese-speaking participant) He just got his, um, booster shot today. And he asked me about mine. I said, I don’t know about that. I don’t know about that. I don’t know. I have very doubts about it. I don’t know. I’m not too happy about it. (U.S.-born Black participant) Moderator: Have you had 3 injections yet or not? Participant: Well, my son has already done 3. I mean, he is going on a business trip, after all, he is afraid. I’ll think about it, maybe, who knows? (Russian-speaking participant)
Direct personal social networks emerged as a strong facilitator of decision making	Trust; Information source (subcode: social networks); Changes over time—views on COVID-19 vaccine;	Well, the first time I heard about it was on TV. So I said to myself, I’m not putting that in my body. I’m not doing that. So one of my nurses, my nieces is a nurse, registered nurse, and she called me and she told me, you don’t have to worry about it? I had it done because it was mandatory in the hospital. We have to have it done. So I said, I’ll think about it. (U.S.-born Black participant) Everybody around me was getting the vaccine I was the only one, if they’re denying and everybody else went and got it and nothing happened to them. And I said, well, let me try then. (Spanish-speaking participant) I had a dialogue with my son who’s also in healthcare. We came to the resolution to try the vaccine. (West-Indian participant)
Immigrant participants shared unique experiences with information and rules	Global perspectives; Rules/comply	Although the [U.S.] government did not say much about the virus at the time, when we went back, we also quarantined ourselves. (Chinese participant) And there are so many discussions! I remember when we were children—we were vaccinated in schools. Doctors and nurses came. And no one asked anyone anything, no one was reading anything on YouTube. If you have to do it, you have to do it. (Russian-speaking participant) Everyone was saying you got these vaccine so quickly. I told you to take some more time to do some more testing and be sure that it’s all right. So... I will try to encourage them to take it…So, as I was telling you, I remember when we had polio, I was in school and they came in to school and they give us the vaccine. Didn’t wait for parents to say no or anything. They came and they did it. And then polio was wiped out for, for some time. We, nobody had polio in a long, long time. (West Indian participant)

## Data Availability

Data are available upon reasonable request to the authors.
